# Nootropic Effects of *C. melo* and *C. lanatus* seed extracts

**DOI:** 10.1155/2020/8823038

**Published:** 2020-12-09

**Authors:** Shahana Wahid, Ali Alqahtani, Rafeeq Alam Khan

**Affiliations:** ^1^Department of Pharmacology, University of Karachi, Karachi, Pakistan; ^2^Department of Pharmacology, College of Pharmacy, King Khalid University, Guraiger, Abha 62529, Saudi Arabia; ^3^Faculty of Pharmacy, Ziauddin University, Karachi, Pakistan

## Abstract

Dementia and related conditions disturb the ability to perform routine life activities prohibiting a person from making appropriate decisions. Seeds of *Cucumis melo* and *Citrullus lanatus* have been investigated extensively for various pharmacological properties; hence, considering the presence of bioactive compounds, it was assumed that these seed extracts may support the functioning of the central nervous system. Thus, the present study was designed to investigate the short-term and long-term memory-enhancing effects of *C. melo* and *C. lanatus* seed extracts in mice by the Morris water maze (spatial learning and memory), stationary rod test, and passive avoidance tests (fear-motivated tests). Ethanol extract of both seeds were prepared by standard procedure and given to animals in the doses of 50 mg/kg, 100 mg/kg, and 200 mg/kg. The results were compared to standard drugs diazepam and imipramine given in the doses of 3 mg/kg and 30 mg/kg, respectively. Extracts of both the seeds were found to possess significant memory and cognition-enhancing effects in mice when tested by passive avoidance, stationary rod, and water maze tests. Results demonstrate memory and cognition-enhancing effects of these extracts which may be due to the presence of bioactive compounds in these seeds.

## 1. Introduction

Dementia and related conditions disturb the ability to perform routine life activities prohibiting a person from making appropriate decisions. Learning and memory evaluation is the most tested paradigm which explores the effect of drugs on certain regions of the brain according to the model selected. Learning is the process of attainment of new memories while memory is the recalling of past learned events. It involves encoding, storage, retrieval, and forgetting [1]. Short-term memory is defined as memory over a short time interval while long-term memory refers to the retention of information for a prolonged period of time. Long-term memory is developed due to periodic repetition of information [2]. The normal learning process depends on neurotransmitters like acetylcholine, dopamine, and 5HT which activate the hippocampus (new learning) and amygdala (fear and emotional memories) and other brain areas such as the primary sensory cortex, visual cortex, and auditory cortex [3]. The loss of memory is greatly accelerated by anxiolytics, sedatives, or substances of abuse which result in amnesia. However, cholinesterase inhibitors and dopamine agonists are used to treat dementia [4].

Alzheimer is a neurodegenerative disease with many neuropsychiatric and cognitive problems causing progressive disability and dementia. The condition is either due to the loss of cholinergic neurons especially in the forebrain or reduction in concentration of acetylcholine. The most hopeful treatment for AD is the use of enzyme acetyl cholinesterase (AChE) inhibitors which mainly produce effects by increasing acetyl choline concentration in the brain. However, drugs which improve memory will have a beneficial effect in Alzheimer's disease (AD). Various plants have been used traditionally for management of many diseases due to the presence of bioactive compounds; thus, extracts from *C. melo* and *C. lanatus* may lead to discovery of new compounds.

*Cucumis melo* seeds are a rich source of minerals; vitamins; and enzyme inhibitors like magnesium, potassium, calcium, and sodium [5]; vitamin A; vitamin C; cucurbitacin A, B, and E [6]; *β*-carotenes; Apo carotenoids; phosphatidylcholine; melanin; cucumisin; and trypsin inhibitors CMeTI-A and CMeTI-B [7], while various types of glycolipids and free fatty acids are also present like oleic and linolenic acids. Antioxidants such as ellagic acid, gallic acid, and caffeic acid are also present in traces [8, 9]. Chromone derivatives like beta-sitosterol, beta-amyrin, and beta-sitosterol-3-O-betaglucopyranoside are also present [10].

*C. melo* has been studied for its antioxidant, gastroprotective, analgesic, anti-inflammatory, antimicrobial, laxative, antigiardial, hepatoprotective, and atherosclerotic properties [11]. There are several studies which show numerous pharmacological properties of *C. melo* seeds like analgesic, anti-inflammatory, antioxidant, anticancer, antidiabetic, antiulcer, diuretic and hepatoprotective effects [12]. These effects were thought to be the presence of various phytochemicals [13]. However, no work has been done on CNS effect of *C. melo* seed extract.

*C. lanatus* is a vitamin C-rich fruit having important phytochemicals and essential amino acids like citrulline, arginine, b-glutamine, and C-aspartic acid [14, 15]. Phytochemicals reported are cucurbitacin E, phenolic compounds, sterols, alkaloids, caprylic acid, capric acid, lauric acid, myristic acid, palmitic acid, stearic acid, oleic acid, linoleic acid, and sterol [16]. Its fruit is utilized for its relaxing and cooling effects.

The seed extract of *C. lanatus* showed antimicrobial [17], antioxidant, antiulcer [18], and anti-inflammatory activities [19]. The seeds of *C. lanatus* have also found to possess potent analgesic, antitussive, antipyretic, anthelmintic, and immune-modulatory properties, while also having antibacterial, gastroprotective, laxative, antigiardial, and hepatoprotective properties [11]. The seeds can be used for reducing the risk of coronary heart disease and cancer, while also possessing diuretic, antimicrobial, and antifungal activities [19]. Despite all these pharmacological activities, little work has been done to explore the CNS effects of *C. lanatus.*

The loss of memory is not only associated with Alzheimer's but also afflicts individuals under stress. Thus, considering the seriousness of the problem and effectiveness of *C. melo* and *C. lanatus* seeds in improving overall CNS functions in the peoples of Southeast Asia over years, it was decided, based on previous pharmacological work, to scientifically evaluate memory-enhancing effects of *C. melo* and *C. lanatus* [11, 15, 16, 18, 19].

## 2. Material and Methods

### 2.1. Ethical Statement

The study was accompanied following the approval from the Board of Advance Studies and Research, University of Karachi. The reference no. 03297/Pharm was granted by the Board on April 20, 2017, to conduct the study. This approval was followed by the permission of the Departmental Ethical Committee, Department of Pharmacology, for the use of animals as per the National Institutes of Health (NIH) guide for the care and usage of laboratory animals [20].

#### 2.1.1. Preparation of Extract

Seeds of *C. melo* and *C. lanatus* were obtained from local medicinal herb sellers, identified by a botanist at the Herbarium, Centre for Plant Conservation, University of Karachi, and deposited as voucher specimen numbers 94494 and 9462, respectively. These seeds after proper washing and drying were milled finely and soaked in 95% ethanol solution for 21 days. The ratio of soaking was 1 kg in 1.5 liters. The mixture was agitated several times during the maceration period. The extract was filtered, air dried, freeze dried, and stored in tightly closed bottles at 4°C until used. The extraction value of *C. melo* and *C. lanatus* was 23.06% and 17.06%, respectively.

### 2.2. Experimental Procedures

The extracts of *C. melo* and *C. lanatus* were administered daily once a day for 30 days. Behavioral experiments were mainly conducted on days 1, 8, and 21.

#### 2.2.1. Passive Avoidance Test

The passive avoidance test is among the quick methods for evaluation of fear aversive memory. The avoidance reaction was determined using an apparatus having light and dark compartments with a grid floor separated by a guillotine door. The black compartment had a grid floor connected to a current supply to give bearable foot shocks. This test was performed in phases of habituation, training, and test sessions. On the 1^st^ and 2^nd^ days of testing, mice were placed in the lighted compartment to habituate for 300 seconds. On the training day, mice were kept in the lighted compartment, facing towards the dark compartment and the guillotine door was opened. When the mice came into the dark compartment, the door was closed and a foot shock (0.6 mA, 0.5 s duration) was delivered through the grid floor [21]. During the trial, the mice were reintroduced into the apparatus after 3 hours, 24 hours, 8 days, and 21 days to evaluate short-term as well as long-term memory. Mice were placed in the lighted compartment again, and the door was kept open to offer free entry into the dark aversive compartment. The latency to reenter into the dark compartment was noted without applying further current stimulus with a cut-off time of 300 s.

#### 2.2.2. Stationary Rod Test

The learning ability was evaluated by stationary rod test having two elevated stainless steel rods with a fixed netted platform. Initially, mice were trained to learn to walk on the elevated rod by keeping the mice at the center of the stationary rod and pushing towards the platform. Animals were given 4 training trials daily for 4 consecutive days, and a single trial of 120 s. After training, DMSO, standard drug, *C. melo*, and *C. lanatus* seed extracts were administered by oral gavage tube for 30 days. Time to reach the platform from the center was noted at day 1 for short-term learning and at the 8^th^ day and the 21^st^ day for long-term memory [22].

#### 2.2.3. Morris Water Maze Test

The water maze test (MWM) is a commonly recognized method for memory testing. It comprises a rectangular water pool (60 × 30 cm) having a fixed central platform (15 × 13 cm) filled by starchy tap water maintained at 22-25°C. During the training period, the platform was visible as the surface of the water was lower, and mice were placed in a pool and permitted to remain on the platform for 10 s. Then, the mice were returned to the home cage during the second-trial interval of 120 seconds. The mice that were not able to explore the platform within 120 s underwent another training session after 10 s interval [23]. Animals were given 4 training trials daily for 4 consecutive days. DMSO, standard drugs, *C. melo*, and *C. lanatus* seed extracts were administered to the mice by oral route after the training trial as per study design, and the mice were tested on the 1^st^day, 8^th^ day, and 21^st^ day. Decrease in time was taken as memory-recalling process improvement [24–26].

#### 2.2.4. Experimental Animals

Few pairs of albino mice BALB/c weighing in the range of 23-29 g were procured from the Hussain Ebrahim Jamal (HEJ) Research Institute of Chemistry, University of Karachi. Animals were then bred at the animal house, Department of Pharmacology, for use in the study. Young mice, weighing 20–28 g, were housed in a cage at 25°C with a controlled alternate 12 h light and dark cycle having no restriction to food and water. Animals were randomly divided into 9 groups having 10 mice in each group (total 90 animals). The control group received 5% DMSO; mice in the standard groups received diazepam 3 mg/kg [27] and imipramine 30 mg/kg [28]; and mice in the test groups received *C. melo* and *C. lanatus* seed extracts at 50, 100, and 200 mg/kg, respectively, for 30 days. After completion of the study, the mice were sacrificed by decapitation.

### 2.3. Statistical Methods

Data analysis was performed by one-way ANOVA utilizing SPSS-20 (Superior Performance Statistical Software) and shown as the mean ± SEM followed by a post hoc Dunnett test to compare values with the control. *P* value < 0.05 was considered as significant while *P* < 0.01 as highly significant.

## 3. Results

### 3.1. Passive Avoidance Test

Table 1 shows the effects of ethanol extract of *C. melo* and *C. lanatus* seeds on memory by passive avoidance test. *C. melo* at 50 mg/kg and 100 mg/kg exhibited highly significant increase in latency time to enter the dark chamber at 3 h, 24 h, 8^th^ day, and 21^st^ day while *C. melo* at 200 mg/kg exhibited highly significant increase in latency time up to the 8^th^ day. *C. lanatus* at 50 mg/kg exhibited significant increase in latency time to enter the dark chamber at 3 h and highly significant increase in latency time at 24 h and 8^th^ day. *C. lanatus* at 100 mg/kg showed highly significant increase in latency time to enter the dark chamber at 3 h, 24 h, 8^th^ day, and 21^st^ day as compare to the control, while the *C. lanatus* at 200 mg/kg group showed significant increase in latency time to enter the dark chamber at 3 h and highly significant increase in latency time at 24 h and 21^st^ day. Latency time to enter in dark compartment was found significantly shorter in mice receiving standard drug diazepam 3 mg/kg on the 8th and 21st days, while in the case of mice receiving imipramine 30 mg/kg, latency time to enter the dark compartment was found significantly longer at 3 h, 24 h, 8^th^ day, and 21^st^ day as compared to the control.

### 3.2. Stationary Rod Test

Table 2 shows effects of ethanol extract of *C. melo* and *C. lanatus* seeds on memory by stationary rod test. *C. melo* seed extracts at 50 mg/kg and 100 mg/kg exhibited highly significant decrease in time to reach elevated platform at 24 h, 8^th^ day, and 21^st^ day as compared to the control.

*C. lanatus* at 50 mg/kg and 100 mg/kg exhibited highly significant decrease in the time to reach the platform at 24 h, 8^th^ day, and 21^st^ day as compared to the control, while *C. lanatus* at 200 mg/kg showed significant increase in the latency time at 24 h and a highly significant decrease in the time to reach the platform at the 8^th^ and 21^st^ days as compared to the control.

### 3.3. Water Maze Test

Table 3 shows the memory-enhancing effects of *C. melo* and *C. lanatus* seed extracts in the water maze test. Shorter time to reach the hidden platform was considered as the index of retrieval of memory. *C. melo* at 50 mg/kg exhibited highly significant decrease in time to reach the platform at 24 h, 8^th^ day, and 21^st^ day as compared to the control. *C. melo* at 100 mg/kg exhibited a significant shorter time to reach the platform at 24 h, while it showed a highly significant decrease in time to reach the platform at the 8^th^ and 21^st^ days as compared to the control. *C. melo* at 200 mg/kg showed significant decrease in the time to reach the platform at 24 h and a highly significant decrease in the time to reach the platform on the 8^th^ day.

*C. lanatus* at 50 mg/kg and 100 mg/kg exhibited a highly significant decrease in the time to reach the platform on the 8^th^ day and significant decrease in the time to reach the platform on the 21^st^ day as compared to the control. *C. lanatus* at 200 mg/kg exhibited a highly significant decrease in the time to reach the platform at 24 h and the 8^th^ day while exhibiting significant decrease in time to reach the platform on the 21^st^ day as compared to the control.

Animals given standard drug diazepam 3 mg/kg showed highly significant increase in the time to reach the platform on the 8^th^ and 21^st^ days while imipramine 30 mg/kg showed highly significant decrease in the time to reach the platform on the 8^th^ and 21^st^ days as compared to the control.

## 4. Discussion

Dementia is a condition which is either produced due to the use of drugs like anxiolytics, sedatives, or neurodegenerative disorders like Parkinson's and Alzheimer or aging [4]. According to Ahmed and coworkers [29], worldwide, 24.3 million people are suffering from dementia with the increment of 4.6 million cases every year making it a major contributor of normal life disability. This emphasizes the need of newer drugs which can improve the memory without adverse effects. Hence, the current study was designed to explore nootropic effects of edible seeds using three different models i.e., the water maze, stationary rod, and passive avoidance tests. In all three models, seed extracts at 50 mg/kg and 100 mg/kg showed memory-enhancing effects as indicated by a significant increase in latency time both at short- and long-term levels as compared to the control. However, at the 200 mg/kg dose, the effects were similar to the control due to increase in passivity.

In the present study, nootropic effects of *C. melo* and *C. lanatus* were evaluated using three different models of memory, i.e., passive avoidance, stationary rod, and water maze tests. Seed extracts of *C. melo* and *C. lanatus* showed an increment in the memory-recalling process for short- and long-term memory as compared to the control and standard in all three models at doses of 50 and 100 mg/kg; hence, it may be suggested that both seeds have cognition enhancement ability. However, at 200 mg/kg doses, effects were similar to the control due to the increment in passivity.

There are several drugs which at high doses may activate sedative activity and passivity just like diazepam. In the treatment of moderate anxiety, the initially selected human dose is 2 mg thrice a day which can be increased depending upon the required anxiolytic effects, while for sleep problems, the starting dose is 5 mg which is almost twice that of the anxiolytic dose which can be increased up to 15 mg.

Memory-enhancing effects of *C. melo* and *C. lanatus* may be due to the presence of cucurbitacin B and cucurbitacin E, since these cucurbitacins are thought to produce neurogenesis and neuroprotective effects [30, 31]. The bioactive compounds in *C. melo* are thought to inhibit *β*-secretase responsible for producing *β*-amyloid [32], which is a toxic protein, causing neuronal loss in selected brain areas, hence producing memory-enhancing effects. Sang-Shin and coworkers reported memory-enhancing effects of the *C. melo* extract at the dose of 100 mg/kg due to inhibition of acetyl cholinesterase; thus, results of the present study are in accordance with previous studies.

Seeds of *C. melo* contain ketogenic amines like tryptophan and lysine [33] which improve cognitive functions. Thaipisuttikul and Galvin [34] reported that *C. lanatus* contains caprylic acid which is thought to bypass the metabolic impairment of energy as identified in AD. Hence, various dietary patterns containing a ketogenic diet are suggested to reduce the neuropathological complications [35]. Furthermore, *C. lanatus* contains L-citrulline which is a precursor of arginine [36]. Citrulline has been supposed to increase energy levels, stimulate immune system, and detoxify ammonia [37].

Citrulline due to its potent hydroxyl free radical scavenging activity acts as an immunomodulator and is supposed to be beneficial in various conditions including multi-infarct dementia. It also enhances NO production and in turn suppresses the risk of myocardial oxidative stress. Hence, these seeds may be effective for prevention and treatment of oxidative stress-induced cardiovascular disease [38].

Evidence suggests that NO also have a role in learning and memory on activation of N-methyl-D-aspartate (NMDA) receptors [39]. NMDA receptors in the brain have been shown to play a vital role in several types of learning [40]. Studies in animals also revealed that blockade of NO synthesis *in vivo* impairs learning behavior [41]. Moreover, L-arginine has been found to decrease lipid peroxidation and improve cognitive function in elderly patients with dementia [42]. Further Yi et al. [43] revealed that L-arginine improves loss of memory due to its effects on neurogenesis.

The findings of the current study are quite significant since they imply memory-enhancing effects on the symptoms of AD, which is a chronic progressive brain disease associated with loss of memory and diminished intellectual capabilities due to degeneration of neurons.

## 5. Conclusion

The study has put forward significant findings regarding CNS-related benefits of *C. melo* and *C. lanatus*, since extracts of these seeds have been effective in improving memory and cognition. It implies that such properties may have a role in reducing amnesia. Moreover, being a dietary component can be used safely in elderly patients to reduce the symptoms of Alzheimer disease and amnesia.

## Figures and Tables

**Table 1:** Effects of *C. melo* and *C. lanatus* seed extracts on memory (passive avoidance).

Groups & doses (mg/kg)	Latency time (sec)			
	3 h	24 h	8^th^ day	21^st^ day
Control	115 ± 34	99 ± 11.6	94 ± 15	55 ± 6.26
*C. melo* 50	274 ± 26^∗∗^	300 ± .0.0^∗∗^	271 ± 29.1^∗∗^	261 ± 28^∗∗^
*C. melo* 100	272 ± 28^∗∗^	258 ± 29^∗∗^	242 ± 29.7^∗∗^	246 ± 28^∗∗^
*C. melo* 200	266 ± 28^∗∗^	289 ± 10.5^∗∗^	259 ± 27.3^∗∗^	111 ± 27
*C. lanatus* 50	236 ± 32^∗^	281 ± 18.5^∗∗^	285 ± 15.2^∗∗^	104 ± 33
*C. lanatus* 100	300 ± 0.0^∗∗^	300 ± 0.0^∗∗^	271 ± 29^∗∗^	222 ± 26^∗∗^
*C. lanatus* 200	226 ± 36^∗^	260 ± 26.7^∗∗^	168.65 ± 36	237 ± 28.5^∗∗^
Diazepam 3	155 ± 65	86 ± 13.5	16 ± 3.1^∗^	32 ± 14^∗^
Imipramine 30	257 ± 43^∗^	245 ± 54.6^∗^	269 ± 31^∗^	209 ± 39^∗^

*n* = 10. Mean ± SEM. ^∗^*P* < 0.05 significant as compared to the control; ^∗∗^*P* < 0.01 highly significant as compared to the control.

**Table 2:** Effects of *C. melo and C. lanatus* seed extracts on memory by stationary rod.

Groups & doses (mg/kg)	Time to reach platform (sec)			
	Learning	24 h	8^th^ day	21^st^ day
Control	12.04 ± 1.0	19.5 ± 1.1	25.9 ± 0.8	26.3 ± 1.1
*C. melo* 50	11.9 ± 0.8	7.6 ± 0.9^∗∗^	5.3 ± 0.3^∗∗^	9.9 ± 1.3^∗∗^
*C. melo* 100	14 ± 1.1	6.7 ± 0.4^∗∗^	8.0 ± 0.7^∗∗^	10.0 ± 1.0^∗∗^
*C. melo* 200	18 ± 1.1	14.8 ± 1.5	12.5 ± 1	15.1 ± 1.2
*C. lanatus* 50	16.5 ± 1.3	6.6 ± 0.5^∗∗^	7 ± 0.6^∗∗^	8.8 ± 1.4^∗∗^
*C. lanatus* 100	12.7 ± 0.5	8.8 ± 0.9^∗∗^	8.3 ± 1^∗∗^	8.7 ± 1.1^∗∗^
*C. lanatus* 200	14.5 ± 1.5	11.1 ± 0.7^∗^	11.6 ± 0.6^∗∗^	9 ± 1.0^∗∗^
Diazepam 3	16.3 ± 0.6	28 ± 5	63.4 ± 8.5^∗∗^	82.9 ± 10.5^∗∗^
Imipramine 30	13.9 ± 1.6	11.9 ± 1.8	8.2 ± 1.5^∗∗^	6.2 ± 0.9^∗∗^

*n* = 10. Mean ± SEM. ^∗^*P* < 0.05 significant as compared to the control; ^∗∗^*P* < 0.01 highly significant as compared to the control.

**Table 3:** Effects of *C. melo* and *C. lanatus* seed extracts on memory by water maze test.

Groups & doses (mg/kg)	Time to reach platform (sec)			
	Learning	24 h	8^th^ day	21^st^ day
Control	14.4 ± 1.5	10.3 ± 0.7	19.0 ± 0.9	20.3 ± 0.7
*C. melo* 50	15.5 ± 1.5	3.4 ± 0.5^∗∗^	3.9 ± 0.5^∗∗^	5.6 ± 0.6^∗^
*C. melo* 100	13.9 ± 1.8	4.7 ± 0.7^∗^	3.3 ± 0.6^∗∗^	6.4 ± 1^∗^
*C. melo* 200	16.3 ± 1.1	5.0 ± 0.9^∗^	6.4 ± 1.0^∗∗^	25.9 ± 3.9
*C. lanatus* 50	9.6 ± 2	5.4 ± 1.04	4.6 ± 0.3^∗∗^	4.4 ± 0.7^∗^
*C. lanatus* 100	7.8 ± 1.4	5.3 ± 0.5	2.6 ± 0.30^∗∗^	3.9 ± 0.6^∗^
*C. lanatus* 200	8.0 ± 2	3.6 ± 0.3^∗∗^	3.5 ± 0.3^∗∗^	5.6 ± 0.7^∗^
Diazepam 3	15.2 ± 0.8	33.8 ± 2.7^∗∗^	36.6 ± 2.9^∗∗^	91.7 ± 13.1^∗∗^
Imipramine 30	13.9 ± 1.6	11.9 ± 1.8	8.2 ± 1.5^∗∗^	6.2 ± 0.9^∗∗^

*n* = 10. Mean ± SEM. ^∗^*P* < 0.05 significant as compared to the control; ^∗∗^*P* < 0.01 highly significant difference as compared to the control.

## Data Availability

The data generated or analyzed in the study is included in this article; its supplementary information is available with the first author which can be made available on request.
